# What Prevents Men Aged 40–64 Years from Prostate Cancer Screening in Namibia?

**DOI:** 10.1155/2016/7962502

**Published:** 2016-01-10

**Authors:** Joseph Kangmennaang, Paul Mkandawire, Isaac Luginaah

**Affiliations:** ^1^Department of Geography and Environment, University of Waterloo, 200 University Avenue West, Waterloo, ON, Canada N2L 3G1; ^2^The Institute of Interdisciplinary Studies, 2201 Dunton Tower, 1125 Colonel By Drive, Ottawa, ON, Canada K1S 5B6; ^3^Department of Geography, Western University, 1151 Richmond Street, London, ON, Canada N6A 5C2

## Abstract

*Objectives*. Although a growing body of evidence demonstrates the public health burden of prostate cancer in SSA, relatively little is known about the underlying factors surrounding the low levels of testing for the disease in the context of this region. Using Namibia Demographic Health Survey dataset (NDHS, 2013), we examined the factors that influence men's decision to screen for prostate cancer in Namibia.* Methods*. We use complementary log-log regression models to explore the determinants of screening for prostate cancer. We also corrected for the effect of unobserved heterogeneity that may affect screening behaviours at the cluster level.* Results*. The results show that health insurance coverage (OR = 2.95, *p* = 0.01) is an important predictor of screening for prostate cancer in Namibia. In addition, higher education and discussing reproductive issues with a health worker (OR = 2.02, *p* = 0.05) were more likely to screening for prostate cancer.* Conclusions*. A universal health insurance scheme may be necessary to increase uptake of prostate cancer screening. However it needs to be acknowledged that expanded screening can have negative consequences and any allocation of scarce resources towards screening must be guided by evidence obtained from the local context about the costs and benefits of screening.

## 1. Introduction

The global burden of cancer continues to rise with about 14.1 million new cancer cases, 8.2 million cancer deaths, and about 32.6 million people living with the disease worldwide (within 5 years of diagnosis) in 2012 [1]. Also, about 57% (8 million) of the new cancer cases, 65% (5.3 million) of the cancer deaths, and 48% (15.6 million) of the 5-year prevalent cancer cases were recorded in less developed countries in 2012 [1, 2]. One of the major contributors to the growth in cancer incidence is prostate cancer, the second most common cancer among men and fourth among both sexes. Although developing countries account for less than 30% of all cases of prostate cancer, they have the highest estimated mortality from the disease [3, 4]. Similarly, even though Sub-Saharan Africa (SSA) has low prevalence of prostate cancer, it has one of the highest estimated incidence of the disease in the world [5, 6].

Although it is already a major public health concern, the burden of prostate cancer in SSA is expected to grow mainly due to growth and aging of population, changing diets, lifestyles, and socioeconomic conditions [5, 7, 8]. Some studies have even argued that the somewhat relatively lower trends in SSA understate the true magnitude of the disease due to low detection rate as many prostate cancer cases go undiagnosed due to lack of medial knowledge, diagnostic facilities, trained health personnel, and prostate-specific antigen (PSA) testing [9–11]. Consequently, prostate cancer is said to be a leading cause of mortality in resource-poor settings [11–14].

While prostate cancer is the most common kind of cancer among men of African descent [3], testing among African men is uncommon. Low uptake of testing is linked to barriers related to access to cancer-related health care, including diagnosis and treatment and general lack of medical information on risk factors. Yet screening can significantly reduce morbidity and death from prostate cancer [11, 14–17]. For instance, prostate cancer screening may lead to early diagnostics and treatment that could reduce the risk of advanced disease, reduce the risk of dying from prostate cancer, and increase life expectancy [16, 18]. Even though the absolute reduction in the risk of death due to prostate cancer screening may be small, the reductions in the risks of metastasis and local tumor progression are substantial if cases are detected early [19]. To evaluate the efficacy of prostate cancer screening, two large randomized trials were initiated in the early 1990s: the European Randomized Study of Screening for Prostate Cancer (ERSPC) in Europe and the Prostate, Lung, Colorectal, and Ovary (PLCO) trial in the United States. The ERSPC trial showed a significant reduction in the risk of death from prostate cancer in the screening group indicating about 21% and about 29% for men that actually were screened after a median follow-up of eleven years [18] and a substantial increase in absolute effect [20]. The second trial (Goteborg trial) showed that, after a median follow-up of 14 years, one center of the ERSPC trial showed a 44% reduction in prostate cancer mortality among men in the screening group and a 56% reduction for men screened at least once [21]. The PLCO reported no reduction in mortality in the screening group, even though there have been concerns of high contamination in the control arm [22, 23]. However, PSA screening is also associated with unfavorable effects. An adverse effect of screening is overdiagnosis [24]—the detection of cancers that would not have been diagnosed during a persons' lifetime if they had not been screened. Psychologically, men with screen-detected prostate cancer may have to live the rest of their lives with the knowledge that they have prostate cancer [24, 25]. Furthermore, those who opt for curative treatment risk living many years with the side effects of treatment, which would otherwise be symptom-free years [24, 26]. Targeted screening, whereby a person with a family history of the disease are screened, would be necessary to reduce incidence of false positives and the burden of overdiagnosis and overtreatment [27]. However, population level testing may be useful in revealing at-risk populations for active surveillance, watchful waiting among patients with clinically confined low-risk PC, and active public health sensitization [28]. The identification of at-risk populations might inform preventive efforts, by advocating for individual lifestyle changes to reduce the incidence of disease. This approach is referred to as the high-risk approach to prevention because it targets high risk individuals and has dominated preventive efforts in developed countries over decades [26, 27]. However others are of the view that a population level strategy that seeks to control the determinants of incidence in the general population as a whole may hold larger promise in terms of health promotion overall [28].

In Namibia, prostate cancer accounts for 44.8 per 100,000 of all cancers among men. It also accounts for 21.2 per 100,000 of overall cancer incidence and 21.5 per 100,000 of all cancer mortalities among adult men. Further, the country has a 5-year prevalence rate of 28 per 100,000 [2]. Despite being the leading cause of cancer-related death among men and second among both sexes, prostate cancer has historically received low public health priority in Namibia. However, this situation has somewhat changed in the recent years, especially following the introduction of the national awareness, screening, and early treatment campaign, aimed at screening men mostly using prostate-specific antigen (PSA) or rectal exam. Based on PSA level or rectal exam findings, a biopsy is conducted. Once diagnosed, patients are recommended to undergo hormonal therapy. The Namibian Cancer Society as well as the Canadian Society recommends that men in their 40s should be screened annually [29, 30]. Despite these laudable efforts, screening rates have been low.

Factors such as inadequate public health infrastructure and other health concerns such as HIV/AIDS and malaria compete for scarce health resources and likely undermine the provision of prostate cancer services. These challenges have similarly been observed in cervical cancer screening in Namibia [31] and in other regions such as South Africa [4]. For example, it has been suggested that factors that contribute to the low levels of screening largely stem from differences in health care access, lack of knowledge and information, and unavailability of early detection services [4, 32, 33]. In the United States, health insurance coverage has been shown to be associated with increased testing, early detection, and effective treatment of cancer cases [10, 34–36], echoing the vital role of health insurance as a major predictor of cancer screening.

Namibia's health insurance (medical aid) scheme relies on government and private not-for-profit organisations to manage health financing. The private not-for-profit organisations mostly provide “high-option” products and extensive coverage for inpatient and outpatient services to their voluntarily registered members under two main schemes referred to as open and closed schemes [37]. The closed scheme limits membership to a particular industry whilst the open scheme sells medical aid policies to any company or individual that wishes to purchase coverage. Each scheme typically offers numerous policies with diverse benefit packages and premiums with the Namibian Financial Institutions Supervisory Authority (NAMFISA) providing oversight and regulatory roles on the activities of these schemes [37]. About 184,000 public servants and their dependents have been enrolled in the government scheme [38]. Overall, approximately 51% of Namibians who are employed in the formal sector have health insurance with roughly 16–18% of total population under medical insurance [38]. This leaves the unemployed citizens and the majority of the population (82%) without health insurance. This creates a situation where they must rely on out-of-pocket payments or seek care from the public sector, where only basic services are delivered largely free of charge and at low quality.

In a comparative study of Canada and United States [39], it was also found that socioeconomic status as measured by education or occupation status played an important role in cancer screening. This implies that even if cancer services were widely available, not all individuals would have equal access to screening services. While there are several studies in Western countries on access to prostate cancer testing and treatment, this is not the case in SSA where only a few studies have hitherto been conducted. Nonetheless, the few studies from the SSA region suggest that, overall, there is low level of public awareness about prostate cancer and associated risk factors [40]. This study contributes to knowledge in this regard by examining the correlates of prostate cancer screening among men aged 40–64 years in Namibia. The results, as we hope, will only influence policy in Namibia and SSA.

## 2. Methods

### 2.1. Data

This study used the 2013 Namibia Demographic and Health Survey (NDHS), a nationally representative dataset collected jointly by the National Statistical bureau and Ministry of Health of Namibia and MEASURE DHS program in Calverton, Maryland, USA. The NDHS is administered face to face to men aged between 15 and 64 years and collected periodically to provide data on basic national demographic and health indicators to guide policy makers, planners, and researchers. It is one of the few national surveys in SSA which has recently introduced a set of indicators on prostate cancer screening in order to assess the prevalence and risk factors in the general population. The current study focuses on a subsample of 1,244 men aged 40 and above.

### 2.2. Measures

The outcome variable of this study, prostate cancer screening, is a binary dependent variable measured with the question “have you ever been examined for prostate cancer?”, coded “0” for no and “1” for yes. The main explanatory variable of the study—health insurance coverage—was constructed from the question “are you covered by health insurance?”, coded “0” for not covered and “1” for covered. To capture the role of capacity for health literacy, the study also included a variable on education “0” for no education, “1” for primary, “2” for secondary, and “3” for higher and whether men discussed family planning issues with a health worker in the last 12 months “0” for no and “1” for yes. The role of health literacy was further explored by the variable tapping into exposure to media in which men were asked as to whether they listen to radio coded “0” for not at all, “1” for often, and “2” for very often or watch television coded “0” for not at all, “1” for often, and “2” for very often. This was important given the use of mass media in the dissemination of medical information in Namibia and other SSA countries. The analysis also examined the mediating effect of socioeconomic status using wealth quintiles. Wealth is a composite index created based on a household's ownership of a number of consumer items which the NDHS deems to be poorest, poorer, middle, richer, and richest quintiles and recoded “0” for poorest and poorer; “1” for middle; and “2” for richer and richest. Demographic variables included in the analysis are age of respondents in 5-year categories, marital status coded “0” for single; “1” for married, and “2” for separated, and religion coded “0” for Catholics; “1” for Protestants; “2” for ELCIN (a type of Christian religion practiced in Namibia); and “3” for other religious groups. Locational factors controlled for include place of residence coded “0” for urban, “1” for rural and geographic region of residence coded “0” for Caprivi, “1” for Erongo, “2” for Hardap, “3” for Kara, “4” for Kavango “5” for Khomas, “6” for Kunene, “7” for Ohangwena, “8” for Omaheke, “9” for Omusati, “10” for Oshana, “11” for Oshikoto, and “12” for Otjozondjupa. The reference categories of all variables are coded “0.”

### 2.3. Analytical Strategy

We used complementary log-log models instead of binary logit model to analyze our outcome variable given the highly uneven split of the outcomes in the dependent variable (see Table 1). Standard logit models are also built under the assumption of independence of respondents, but the NDHS has a hierarchical structure with respondents nested within survey clusters with a possibility of biasing our standard errors. To avoid bias in the standard errors and parameter estimates, a random effects regression analysis that corrects for these biases was employed using GLLAMM in Stata (see [41–44]). Sample characteristics of our dependent and main independent variables with some selected variables are depicted in Table 1.  Table 2 presents our bivariate associations between our dependent variable (prostate cancer screening) and each of the independent variables. This is followed by two multivariate models presented in Table 3.

## 3. Results

### 3.1. Univariate Analysis

Our findings indicate that only 16% of men reported ever screening for prostate cancer in Namibia. About 32% of our sample reported having health insurance and only 5% of men reported ever discussing family planning issues with a health worker in the last 12 months before the survey. About 39% of men had a secondary education, 72% reported being married, with a mean age of 49, and about 41% of the sample were identified with the ElCIN religion. The distribution of men with regard to place of residence was near even between urban and rural areas, 49% of men residing in urban areas and approximately 51% in rural areas. About 50% of men were within the richest and richer wealth quintiles whilst about 31% were in the poorest and poorer categories. The middle wealth quintile accounted for about 20% of the sample.

### 3.2. Bivariate Analysis

The results of the bivariate logistic models are reported in Table 2. Men with health insurance coverage (OR = 6.77, *p* = 0.01) were highly likely to undergo screening for prostate cancer compared to men with no insurance coverage. Similarly, compared to men who reported not discussing reproductive issues with a health personnel in the 12 months prior to the survey, men who reported such a discussion (OR = 1.67, *p* = 0.01) were more likely to report testing for prostate cancer. Men in the age categories, 50–54 (OR = 1.87, *p* = 0.01), 55–59 (OR = 2.08, *p* = 0.01), and 60–64 (OR = 2.10, *p* = 0.01), were more likely to test for prostate cancer compared to men in the 40–44 age category. Relative to Catholics, men who identify with Protestant Christian denominations (OR = 1.71, *p* = 0.05) and other religions (OR = 2.14, *p* = 0.01) were more likely to report screening for prostate cancer. Currently married (OR = 3.85, *p* = 0.01) and separated men (OR = 2.83, *p* = 0.01) were more likely to report testing compared to never married men. Men residing in rural Namibia were less likely (OR = 0.49, *p* = 0.01) to test for prostate cancer compared to their urban counterparts. Regionally, residents in Erongo (OR = 2.49, *p* = 0.10), Karas (OR = 3.00, *p* = 0.05), Khomas (OR = 2.63, *p* = 0.10), and Otjozondjupa (OR = 2.44, *p* = 0.10) were more likely to test for prostate cancer compared to men resident in Caprivi region. Socioeconomically, men in the richer (OR = 4.03, *p* = 0.01) and richest (OR = 13.61, *p* = 0.01) wealth categories were significantly more likely to test for prostate cancer compared to poorer men.

### 3.3. Multivariate Analysis

Two multivariate results are presented in Table 3. In the first model we estimated the effects of health insurance, knowledge, and access to information on men's decision to test for prostate cancer. Like in the bivariate analysis, men with health insurance coverage (OR = 4.11, *p* = 0.01) were more likely to be examined for prostate cancer compared to those uninsured. Also, men with secondary education (OR = 4.03, *p* = 0.01) or higher (OR = 8.13, *p* = 0.01) were more likely to have been screened for prostate cancer compared to men with no formal education.

The second model controls for demographic and socioeconomic variables. We found that the association between health insurance coverage and prostate cancer testing attenuated after adjusting for socioeconomic and demographic variables but remained significant and robust. Other variables associated with screening included level of education, age of respondent, contact with health personnel, region of residence, and wealth category. Compared to men without formal education, men with secondary (OR = 3.32, *p* = 0.01) or higher (OR = 6.34, *p* = 0.01) level of education were all more likely to test for prostate cancer. Remarkably, the findings suggest a steady gradient across successive levels of education, where men with higher than secondary level of education are more like to test for prostate cancer than men with secondary education, who are more likely to test than men with primary education, who in turn are more likely to test for cancer than men without formal education. Like in the bivariate analysis, men who reported having contact with a health worker in the 12 months prior to the survey (OR = 2.02, *p* = 0.05) were more likely to report testing for prostate cancer compared to those without such contact. As is the case with education, there is steady gradient across age where men in a given age group are more likely to test for prostate cancer than men in the age category immediately below them. Thus, compared to the 40–44 age category, men aged between 50 and 54 (OR = 2.05, *p* = 0.01); 55 and 59 (OR = 2.06, *p* = 0.01); and 60 and 64 (OR = 3.30, *p* = 0.01) were all more likely to test. Also, male residents in Ohangwena (OR = 5.10, *p* = 0.01) were more likely to test for prostate cancer compared to male residents in Caprivi, the poorest and underserved region in Namibia. In contrast to men in the poorest wealth quintile, those in the richer (OR = 2.41, *p* = 0.10) and richest (OR = 4.95, *p* = 0.01) wealth quintile were more likely to test for prostate cancer.

## 4. Discussion

We examined the determinants of prostate cancer screening among men of 40 years and over, considered to be the age group at risk of prostate cancer. Our findings show that Namibian men with health insurance coverage, having access to information, having contact with health workers, and residing in richer and richest wealth quintiles, were more likely to screen for prostate cancer. The effect of health insurance on testing for prostate cancer remained robust even after controlling for access to information and socioeconomic and demographic factors, suggesting the disproportionate influence that having insurance coverage might have on an individual's access to cancer screening. This particular finding is generally consistent with the literature on the effect of insurance coverage on health utilisation in different places [10, 31, 35]. One key explanation might be that in Namibia health insurance coverage has a significant potential to reduce out-of-pocket health expenses, increase the frequency of hospital visits and quality of interacting with doctors, and reduce payment for health care at the point of service, including screening for prostate cancer.

Invariably, given the relative contribution of insurance coverage and wealth to prostate cancer screening in this context, it means that the poor face a dual burden of poverty and inequity in health access. Hence, the poor are more likely to be uninsured and are also more likely to face barriers to preventive information on prostate cancer screening. This may be due to access disparities among insured and uninsured individuals, often rooted in income inequalities [45] that may translate into health inequalities in the area of prostate cancer screening. The majority of Namibians rely on low-quality health services provided by the public health system which is already overburdened by the HIV/AIDS epidemic [46]. Overall, only 51% of Namibians employed in the formal sector are covered by health insurance, with only 16–18% of the total population under medical insurance [38]. This leaves the unemployed and majority of the population (82%) without health insurance. These uninsured individuals may have to rely on out-of-pocket payments or to seek care from the public sector, where only basic services are delivered largely free of charge and at low quality [38]. This suggests that the adoption of a universal health insurance scheme that ensures equity may improve testing or screening levels in Namibia. However, we must also acknowledge that universal insurance coverage would not automatically result in equitable access to prostate cancer screening unless efforts are made to improve preventative health information and the availability and accessibility to health services.

An interesting finding of this study is the positive relationship between discussing health issues with a health worker and screening for prostate cancer. This suggests that the appropriate promotion of prostate cancer screening through health workers will be useful to encourage men to test especially in a context where reproductive health services have historically been directed at women. There is the need to push for more openness and awareness in order to promote dialogue between health professionals and men on relevant issues around prostate cancer and encourage them to screen for prostate cancer. Our results are consistent with those of other studies which have reported that individuals who make regular visits or are in regular contact with health worker(s) tend to be better informed about health issues, are familiar with medical settings, are more receptive to medical advice, and are more likely to undergo testing [47, 48]. The significant association between men discussing health issues and screening for prostate cancer reflects more positive attitudes and social motivation toward learning about prostate cancer and engaging in healthy behaviours that may lead to early detection and prevention of the disease [47]. Thus, the finding especially draws attention to the need for increased emphasis on promoting awareness about prostate cancer in order to equip the public with relevant knowledge and encourage men to adopt preventive behaviours including screening. The positive relationship (steady gradient) between education and screening for prostate cancer might also reflect positive attitudes where educated men who are most likely to be aware of or comprehend health risks levels are more likely to take appropriate actions such as testing.

The progressive association between age and testing for prostate cancer may be a reflection of more positive behaviours to learn about risk factors and willingness to adopt preventive measures such as screening in order to seek treatment. This particular finding is generally consistent with other studies that have singled out age as one of the widest known risk factors for developing prostate cancer alongside ethnicity and race [25, 49, 50].

The positive association between wealth and testing for prostate cancer is noteworthy. The richer and richest categories were more likely to report testing for the disease, reemphasising the notion that it is mostly those who have the financial means to overcome barriers to health care services. This is similarly the case in the context of health insurance coverage where the richer and richest tend to have better access to prostate cancer testing. The relatively low likelihood of testing among the poor highlights the issue of socioeconomic inequalities to cancer screening and underscores the kinds of barriers that poor people face in terms of access to testing. Since testing is a gateway to treatment, the findings of this study also suggest potential socioeconomic disparities in morbidity and mortality from cancer in Namibia. Furthermore, even though prostate cancer screening is generally low in Namibia, the findings of this study suggest existence of wide geographical variations in terms of screening. For instance, residents in Ohangwena region were more likely to screen for prostate cancer, compared to Caprivi, one of Namibia's poorest and underserved regions [30]. Namibia is a vast country and in such remote regions, such as Caprivi, up to 60% of the population live more than 5 km from the nearest health facility [51]. Addressing these barriers to screening will prove useful for the government's efforts to curb prostate cancer incidence. However, despite the potential public health benefits of screening, in setting such as Namibia, screening outcomes are likely to be poorer and even when correctly diagnosed patients may not receive treatment due to lack of appropriate resources. It is therefore important for public health officials and policy makers to obtain local context evidence of screening as well as weigh the cost and benefits of expanded prostate cancer screening to other public health interventions.

This study has some limitations. First, due to the cross-sectional nature of the dataset, we are unable to make causal linkages between prostate cancer screening and any of our independent variables. Also, due to the self-reported nature of the data, some biases may have been introduced into the data during data collection as men are more likely to provide socially satisfactory responses and the NDHS could not physically validate these responses. We do also acknowledge that GLOBOCAN data on mortality are projections and may overstate or understate the burden of prostate cancer in Namibia. Furthermore, even though prostate cancer is deserving public health attention, it should be noted that there still remain other more common noncancer causes of mortality and that knowing one's prostate cancer status will not necessarily prevent death. Such considerations should be factored in when prioritizing public health policies in such limited resources settings that have to prioritize their objectives. To a large extent our results are generalizable to other resource-poor countries in Sub-Saharan Africa, even though one must not lose sight of the contextual influence of culture, norms, health behaviours, and the political support of Namibian government in prioritizing population based screening.

In conclusion, this paper has examined the determinants of prostate cancer among men aged 40–64 years in Namibia. The significant role played by health insurance coverage in influencing screening highlights the need for a national health insurance strategy that ensures equity in health access, especially screening for cancer. Currently, Namibia does not have a universal health insurance policy although discussions are currently underway to introduce a national scheme to reduce out-of-pocket health expenses and inequities in access to health services. It is hoped that this may impact positively on health care utilisation including prostate screening. We also urge that for such a scheme to be effective in increasing screening for prostate cancer, it has to be accompanied by a strong health promotion campaign to promote the public awareness about the disease. The study also points to the role played by regular contact with health workers in promoting testing for prostate cancer among men, underscoring the need for the government to reduce barriers that make it difficult for people to get in touch with health personnel or to have a regular doctor. It also suggests the need for a cultural shift that would promote more dialogue on men's reproductive health issues in a context where women have traditionally been the subject of such debates. The current recommendations for prostate cancer screening are more appropriate for developed country contexts as they have the resources and technical expertise to handle the burden of prostate cancer. In a resource limited setting such as Namibia, outcomes are likely to be poorer and there is also the strong likelihood that many patients that would be correctly diagnosed may not receive treatment due to lack of appropriate resources. This is especially the case in Namibia where, due to a lack of previous testing, the national rollout of screening is likely to uncover many cases of advanced prostate cases which may be difficult to treat. As a public health consideration, the Namibian government should carefully consider likely benefits from the national screening program with respect to its capacity to provide appropriate care for those who test positive. Those making decisions about commitment of public health resources need to weigh the costs associated with prostate cancer against those of other public health interventions, such as HIV/AIDS, malaria, or even cervical cancer, whose diagnostics and interventions are relatively low cost. Cervical and breast cancer are also other important and competing public health problems in Namibia.

## Highlights

- Screening for prostate cancer remains low in Namibia with only 16% of men reporting having ever tested.
- Men with health insurance and those who discuss their health issues with a professional were more likely to screen for prostate cancer; the findings suggest that expanding health insurance coverage together with prostate cancer screening education could improve the outcomes.
- The study contributes to the current field of knowledge of prostate cancer testing among resource-poor populations given the high risk of prostate cancer within these settings.

## Figures and Tables

**Table 1:** Sample characteristics.

Variable	Frequency (%)
Ever tested for prostate cancer	
No	1,044 (83.92)
Yes	200 (16.08)
Health insurance (ref: none)	
No	841 (67.60)
Yes	403 (32.40)
Education (ref: none)	
No formal education	226 (18.17)
Primary	420 (33.76)
Secondary	481 (38.67)
Higher	117 (9.41)
Discussed health issues with health worker in the last 12 months	
No	1,185 (95.26)
Yes	59 (4.74)
Frequency of reading newspapers	
Not at all	1,000 (80.39)
Often	145 (11.66)
Very often	99 (7.96)
Frequency of listening to radio	
Not at all	178 (14.31)
Often	274 (22.03)
Very often	792 (63.67)
Age of respondent (mean)	49
Marital status	
Single	239 (19.21)
Married	899 (72.27)
Separated	106 (8.52)
Religion	
Catholic	298 (23.95)
Protestants	203 (16.32)
ELCIN	515 (41.40)
Others	228 (18.33)
Region of residence	
Caprivi	58 (4.66)
Erongo	150 (12.06)
Hardap	127 (10.21)
Karas	126 (10.13)
Kavango	76 (6.11)
Khomas	99 (7.96)
Kunene	89 (7.15)
Ohangwena	48 (3.86)
Omaheke	126 (10.13)
Omusati	76 (6.11)
Oshana	61 (4.90)
Oshikoto	138 (11.09)
Otjozondjupa	
Place of residence	
Urban	615 (49.44)
Rural	629 (50.56)
Wealth	
Poorest	172 (13.83)
Poorer	215 (17.28)
Middle	248 (19.94)
Richer	304 (24.44)
Richest	305 (24.52)

**Table 2:** Bivariate analysis of prostate cancer screening (complementary log-log).

Variable	OR (SE)
Health insurance (ref: none)	
Yes	6.77 (1.172)^*∗∗∗*^
Education (ref: none)	
Primary	2.43 (.976)^*∗∗*^
Secondary	6.75 (2.556)^*∗∗∗*^
Higher	22.64 (9.119)^*∗∗∗*^
Discussed health issues with health worker in the last month (ref: none)	
Yes	2.51 (.746)^*∗∗∗*^
Listen to radio (ref: none)	
Often	0.38 (.125)^*∗∗∗*^
Very often	0.58 (.196)
Watch television (ref: none)	
Often	1.45 (.435)
Very often	1.49 (.391)
Age of respondent (ref: 40–44)	
45–49	1.38 (.312)
50–54	1.87 (.443)^*∗∗∗*^
55–59	2.08 (.522)^*∗∗∗*^
60–64	2.10 (.556)^*∗∗∗*^
Marital status (ref: single)	
Married	3.85 (1.141)^*∗∗∗*^
Separated	2.83 (1.114)^*∗∗∗*^
Religion (ref: Catholic)	
Protestants	1.71 (.455)^*∗∗*^
ELCIN	1.19 (.272)
Others	2.14 (.518)^*∗∗∗*^
Region of residence (ref: Caprivi)	
Erongo	2.49 (1.331)^*∗*^
Hardap	2.08 (1.150)
Karas	3.00 (1.616)^*∗∗*^
Kavango	1.15 (.727)
Khomas	2.63 (1.466)^*∗*^
Kunene	0.56 (.381)
Ohangwena	2.68 (1.660)
Omaheke	1.46 (.833)
Omusati	0.66 (.451)
Oshana	1.23 (.789)
Oshikoto	1.08 (.694)
Otjozondjupa	2.44 (1.324)^*∗*^
Place of residence (ref: urban)	
Rural	0.49 (.091)^*∗∗∗*^
Wealth (ref: poorest)	
Poorer	1.16 (.581)
Middle	1.74 (.796)
Richer	4.03 (1.682)^*∗∗∗*^
Richest	13.61 (5.498)^*∗∗∗*^

Standard errors are in parenthesis. ^*∗∗∗*^ *p* < 0.01, ^*∗∗*^ *p* < 0.05, and ^*∗*^ *p* < 0.1.

**Table 3:** Factors associated with prostate cancer screening (complementary log-log).

Variable	Model (1)	Model (2)
Health insurance (ref: none)		
Yes	4.11 (.787)^*∗∗∗*^	2.95 (.620)^*∗∗∗*^
Education (ref: none)		
Primary	1.98 (.877)	2.08 (.927)
Secondary	4.01 (1.568)^*∗∗∗*^	3.32 (1.392)^*∗∗∗*^
Higher	8.13 (3.450)^*∗∗∗*^	6.34 (2.915)^*∗∗∗*^
Discussed health issues with health worker in the last month (ref: none)		
Yes	1.54 (0.455)	2.02 (.611)^*∗∗*^
Listen to radio (ref: none)		
Often	0.91 (.379)	0.96 (.404)
Very often	1.10 (.473)	0.99 (.440)
Watch television (ref: none)		
Often	1.12 (.338)	1.39 (.445)
Very often	0.94 (.250)	0.83 (.232)
Age of respondent (ref: 40–44)		
45–49		1.24 (.278)
50–54		2.05 (.504)^*∗∗∗*^
55–59		2.06 (.532)^*∗∗∗*^
60–64		3.30 (.990)^*∗∗∗*^
Marital status (ref: single)		
Married		1.56 (.476)
Separated		1.87 (.737)
Religion (ref: Catholic)		
Protestants		1.02 (.275)
ElCIN		1.18 (.271)
Others		1.12 (.278)
Region of residence (ref: Caprivi)		
Erongo		1.25 (.677)
Hardap		1.33 (.750)
Karas		1.71 (.920)
Kavango		1.52 (.958)
Khomas		1.18 (.661)
Kunene		0.82 (.565)
Ohangwena		5.10 (3.222)^*∗∗∗*^
Omaheke		1.29 (.734)
Omusati		0.47 (.320)
Oshana		0.89 (.550)
Oshikoto		1.03 (.653)
Otjozondjupa		2.11 (1.159)
Place of residence (ref: urban)		
Rural		1.51 (.327)
Wealth (ref: poorest)		
Poorer		1.12 (.586)
Middle		1.25 (.628)
Richer		2.41 (1.186)^*∗*^
Richest		4.95 (2.613)^*∗∗∗*^
Random effect		
Variance at the cluster level	2.106 (.353)^*∗∗∗*^	1.56 (.416)^*∗*^
Constant	0.021 (.009)^*∗∗∗*^	0.003 (.003)^*∗∗∗*^

Observations	1,244	1,244

Standard errors are in parenthesis. ^*∗∗∗*^ *p* < 0.01, ^*∗∗*^ *p* < 0.05, and ^*∗*^ *p* < 0.10.
